# RAN MODULATES ALLOSTERIC CROSSTALK BETWEEN IMPORTIN β SURFACES

**DOI:** 10.21203/rs.3.rs-6449265/v1

**Published:** 2025-04-25

**Authors:** Ying-Hui Ko, Fenglin Li, Stephanie Suinn, Junwei Li, Chun-Feng David Hou, Ravi K. Lokareddy, Gino Cingolani

**Affiliations:** 1 Dept. of Biochemistry and Molecular Genetics, The University of Alabama at Birmingham, 1825 University Blvd, Birmingham, AL 35294, USA; 2 Dept. of Biochemistry and Biophysics, Perelman School of Medicine, University of Pennsylvania, Philadelphia, PA 19104, USA; 3 Department of Chemistry and Chemical Biology, Rutgers, the State University of New Jersey, Piscataway, NJ, 08854, USA

## Abstract

A cellular gradient of the GTPase Ran orchestrates the movement of import and export complexes through the Nuclear Pore Complex (NPC). Ran-GTP modulates two essential activities of importin β for nuclear import. On one hand, it reduces the avidity of importin β for phenylalanine-glycine-rich nucleoporins (FG-nups), facilitating the passage of import complexes through the permeability barrier; on the other hand, it disassembles import complexes, releasing the import cargo into the nucleus. The precise mechanisms by which Ran-GTP modulates importin β activities remain hypothetical. Leveraging cryogenic electron microscopy (cryo-EM) single particle analysis, in this paper, we describe four distinct conformational states of importin β in complex with binding effectors encountered during an import reaction, specifically IBB-cargos, FG-repeats, Ran-GTP, and Ran-GTP:RanBP1. Comparing these four states enables us to decipher the conformational landscape of importin β without interference from crystallization agents and lattice forces. By correlating structural data with biochemical activities, we find that Ran-GTP constrains the solenoid structure of importin β, closing four high-affinity FG-binding pockets and displacing import cargos through allosteric crosstalk between the concave and convex surfaces. We propose that this allosteric mechanism is relevant to other β-karyopherins involved in nuclear import.

## INTRODUCTION

Importin β is the prototypical nuclear import receptor^1–4^ and the founding member of a family of transport receptors known as β-karyopherins^5^. Importin β binds import cargos directly or through the adaptor protein importin α, which exists in seven isoforms in humans^6^. In the classical nuclear import pathway, cytoplasmic cargos containing a Nuclear Localization Signal (NLS) assemble into a trimeric importin β:α1:NLS-cargo complex, which docks to the Nuclear Pore Complex (NPC) and moves through the NPC channel due to importin β avidity for phenylalanine-glycine-rich nucleoporins (FG-nups)^7,8^. The chemical structure of the NPC inner channel is unknown, but ample evidence suggests FG-nups form a hydrogel that constitutes a selective permeability barrier^9^. The small GTPase Ran, enriched as Ran-GTP in the nucleus and Ran-GDP in the cytoplasm^10,11^, facilitates the import reaction by influencing the NPC permeability^12^. Ran-GTP binds importin β with high affinity, releasing the NLS-cargo in the nucleus. It also decreases the affinity of importin β for FG-nups, preventing importin β from stalling and thus clogging the NPC^13–16^. Importin β fragments that bind to the NPC but do not associate with Ran-GTP irreversibly clog the pore, inhibiting NLS-dependent protein import^17^.

Ran-GTP influences the permeability of the importin β:α1:NLS-cargo complex as it moves through the NPC^18^. Typically, large cargos require higher levels of Ran-GTP to navigate the NPC^19,20^, and Ran-GTP primarily regulates the cargo’s exit from the NPC^18^. Without Ran-GTP, cargos can enter and explore the entire NPC, but they are roughly 100 times more likely to exit the NPC on the cytoplasmic side, thus failing to reach the nucleus.^18^. NLS-cargos bound to importin α1 require Ran-GTP and the export factor CAS^21^ to dissociate on the nucleoplasmic side of the NPC. In contrast, importin α1:NLS-cargo complexes that do not dissociate at the NPC are returned to the cytoplasm^21^. Ran Binding Protein 1, or RanBP1, is another key player in the import reaction. It exists as a soluble, 23 kDa protein located in the cytoplasm due to a strong Nuclear Export Signal (NES)^22^, although some reports suggest that the protein may shuttle between the cytosol and the nucleus^23–25^. Four conserved RanBP1-like domains also exist in the filamentous nucleoporin Nup358^26–28^ at the cytoplasmic periphery of the NPC. A crystal structure revealed that RanBP1 binds Ran-GTP via a tight embrace^29^. The primary function of RanBP1 is to facilitate GTP hydrolysis by RanGAP1, countering the inhibitory activity of transport factors. Without RanBP1, the nanomolar binding affinity of Ran-GTP with importin β inhibits RanGAP1-dependent GTP hydrolysis. However, in the presence of RanBP1, the trimeric importin β:Ran-GTP:RanBP1 complex becomes susceptible to GTP hydrolysis, which additionally requires the importin β binding (IBB) domain of importin α1 in vitro.^30,31^.

Structural and computational studies have been crucial in deciphering the organization, binding interactions, and conformational dynamics of importin β. Several crystal structures have revealed that importin β adopts a solenoid architecture formed by 19 HEAT repeats^32^, organized into two functionally polarized surfaces. Importin β inner (concave) surface, formed by HEAT helix B, binds Ran-GTP^33^ and the IBB domain^34^ of the adaptor importin α but can also associate directly with nonclassical cargos^35–37^. Importin β outer (convex) surface, formed by A helices, binds FG-nups^38^. A crystal structure of a fragment of importin β (res 1–442) bound to an FG-rich fragment of nucleoporin Nsp1p reveals two binding sites, one primary site on the A helix of HEAT 5 and another on HEAT 6^39^. This FG-binding pattern was also observed in a structure of Kap95p, the yeast homolog of human importin β, bound to Nup1p^40^. Furthermore, a crystal structure of Kap95p bound to human Ran-GTP^41^ revealed an open conformation of Kap95p, in which the C-terminal domain moves away from the closed solenoid seen in the complex with the IBB domain. Based on the conformation of Kap95p observed in this crystal structure, it was proposed that Ran-GTP displaces cargo by inducing a change in the helicoidal pitch of importin β, which opens its concave surface and disassembles the import complex. However, it remains unclear, and is not explained by the above model, how Ran-GTP releases FG-nups from importin β during active transport.

Using cryogenic electron microscopy (cryo-EM) single particle analysis (SPA), we determine five structures of importin β in a frozen-hydrated state bound to IBB-cargos, FG-nups, Ran-GTP, and Ran-GTP:RanBP1. This comparative structural analysis enables us to decipher the conformational plasticity of importin β, overcoming the limitations of previous crystal structures. Our work clarifies how the GTPase Ran-GTP alters the structure of importin β, regulating its binding affinities for cargos and FG-nups.

## RESULTS

### Cryo-EM structure of importin β bound to IBBs and FG-nup

**Figure 1.**
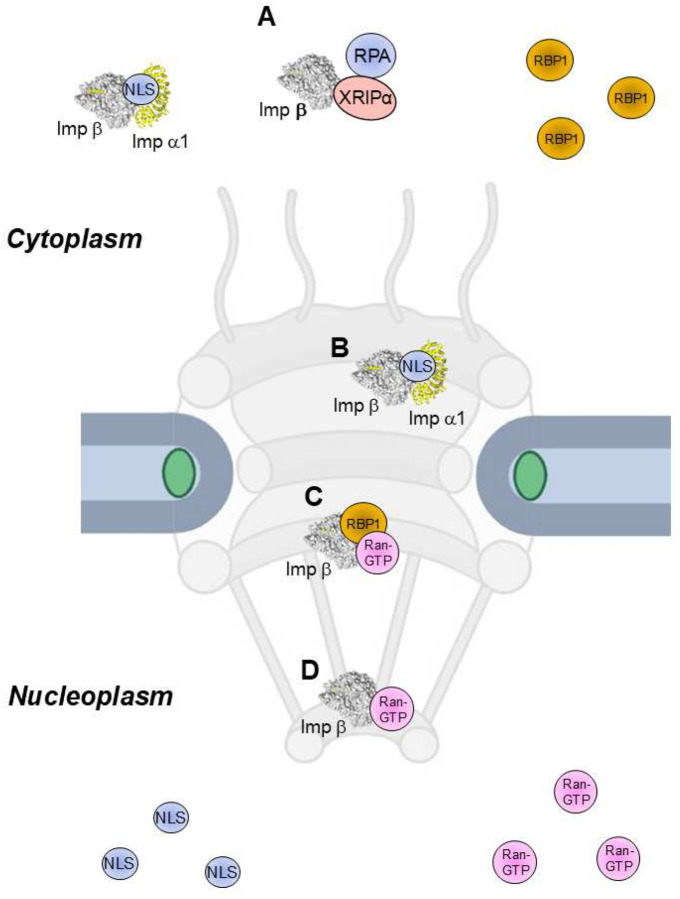
Cartoon representation of importin β complexes involved in nuclear import. (**A**) Classical (importin β:α1:cNLS-cargo) and nonclassical (importin β:XRP1α:RPA) import complexes are formed in the cytoplasm. (**B**) A classical import complex binds to high-affinity FG-nups within the NPC (importin β:αIBB:FG). Complexes of importin β with Ran-GTP:RanBP1 (**C**) and Ran-GTP (**D**) form inside the NPC.

Molecular dynamics simulations^38,42–45^, Förster resonance energy transfer (FRET)^45^, small-angle X-ray scattering (SAXS)^43^, crystallographic^32,36,37,46^, and biochemical^47^ data provided compelling evidence that importin β undergoes large structural fluctuations during nuclear transport. However, most importin β structures have been obtained in the crystalline state in the presence of dehydrating agents that can distort importin β flexible solenoid structure and potentially introduce artifacts^37,46,48^. This study aimed to investigate the structure and conformational changes that importin β undergoes during nuclear import under *quasi-*physiological conditions without potential crystallization-induced artifacts. We formed five complexes of importin β that form during an import reaction. First (Fig. 1A), we assembled importin β with importin α1 IBB (αIBB) and XRIPα IBB (xIBB), an adaptor protein involved in the nuclear import of replication protein A (RPA)^49^. Importin β:αIBB and importin β:xIBB are proxies for cytoplasmic importin β assemblies found in both classical and nonclassical import complexes. Second (Fig. 1B), we added an excess of the FxFG-rich nucleoporin Nsp1^39^ to importin β:αIBB to mimic a classical import complex bound to high-affinity FG sites within the NPC central channel. Third (Fig. 1C, D), we assembled two complexes of importin β with Ran-GTP: the heterodimeric importin β:Ran-GTP complex, which represents the final state of an import reaction presumably formed at the NPC basket, and a trimeric importin β:Ran-GTP:RanBP1 complex that was first identified nearly three decades ago^30,50,51^. All importin β complexes were vitrified and imaged using a 300 kV Krios microscope. Five cryo-EM reconstructions were obtained using SPA, achieving maximal resolutions between 2.6 and 3.4 Å (Table 1, **Supplementary Fig. 1**). The corresponding density maps allowed us to build accurate atomic models of importin β bound to different cell effectors, which were refined to a comparable map-to-model correlation coefficient (CC) (Table 1).

### αIBB and xIBB stabilize distinct importin β conformations

**Figure 2.**
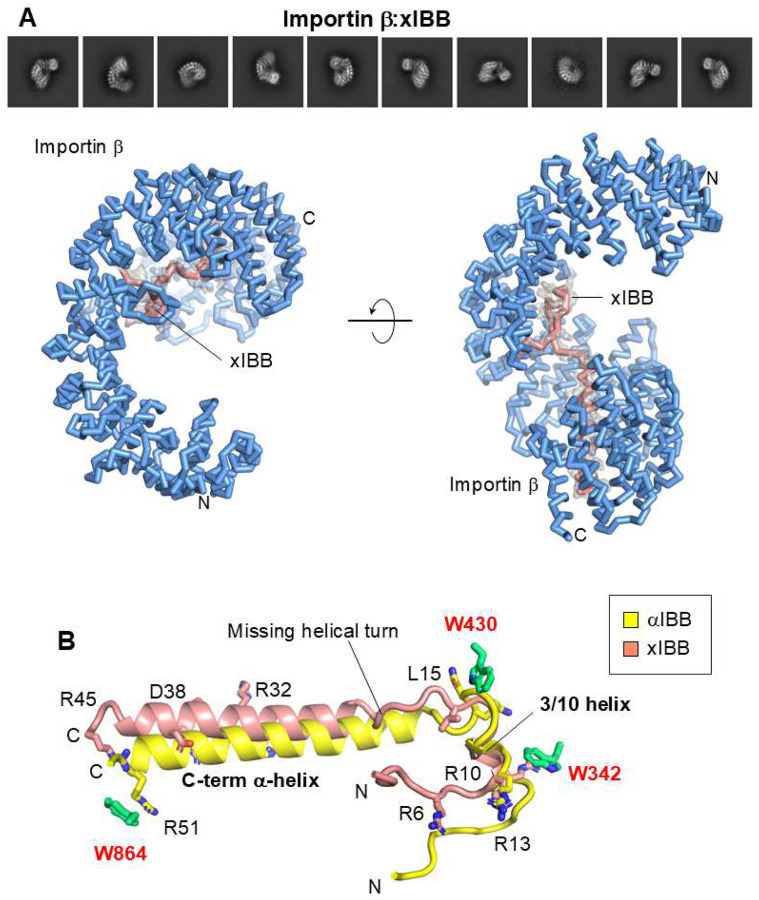
Cryo-EM structure of the importin β:xIBB complex. (**A**) Representative 2D class averages (top) and the 3D structure of the importin β:xIBB complex (bottom). Importin β is shown in blue, while xIBB is depicted in salmon. The final 3.4 Å cryo-EM density (shown in gray) for xIBB is overlaid on the refined model. (**B**) Structural comparison of xBB to αIBB, colored salmon and yellow, respectively. The figure was generated by superimposing the importin β:xBB and importin β:αIBB complexes, displaying only the relative positions of the two IBBs. Importin β tryptophan residues (W343, W430, and W864) that bind to IBB residues are shown in green.

We reconstructed importin β bound to the αIBB domain at 3.3 Å resolution (**Supplementary Figs. 1A** and **2A**, Table 1). This structure, determined in a frozen-hydrated state, revealed significant variations compared to the crystal structure of the importin β:αIBB complex (RMSD ~4.7 Å)^32^, especially in the C-terminal HEATs 11–19 (**Supplementary Fig. 2B**). The N-to-C distance of the X-ray model is 11.5 Å shorter than the cryo-EM structure, suggesting that the constrained, snail-like conformation of importin β:αIBB crystallized at neutral pH^32^ represents a non-physiological state. After establishing that cryo-EM is the preferred methodology for studying importin β curvature, we determined a cryo-EM reconstruction of importin β in complex with xIBB, which we refined to a resolution of 3.4 Å. (**Supplementary Fig. 1B,** Table 1). This new structure revealed an elongated solenoid tightly bound to a ~60 Å long peptide, with importin β spanning ~110 Å (Fig. 2A). The xIBB exhibited strong density for residues 1–45 that contact the concave surface of importin β between HEATs 6–19. A comparison of the two cryo-EM structures of importin β in complex with xIBB and αIBB (solved at comparable resolutions) revealed systematic variations in the solenoid conformation (RMSD ~6.1 Å). The N-to-C distance of importin β bound to xIBB is 9 Å longer than that in complex with αIBB. The two IBBs are highly basic (isoelectric point of ~11) and exhibit a similar topology when bound to importin β (Fig. 2B), consisting of a long C-terminal α-helix and an unstructured N-terminal moiety. However, xIBB is slightly shorter: its C-terminal α-helix spans six turns of a helix (res. 20–45), compared to seven turns of the αIBB. Both IBBs contain an N-terminal 3/10 helix spanning residues R13-F17 and P8-Y13 in αIBB and xIBB, respectively. The bonding pattern is similar but not identical, primarily driven by electrostatic interactions. xIBB forms five salt bridges, seven hydrogen bonds, and 123 non-bonded contacts with importin β, which is significantly fewer than αIBB, where the binding interface with importin β is stabilized by eight salt bridges, ten hydrogen bonds, and 112 non-bonded contacts. Three tryptophan residues in importin β, W342, W430, and W864^52^ (Fig. 2B) play a crucial role in IBB recognition, by making cation-π interactions with IBB arginine and lysine residues. Thus, cryo-EM reconstructions of importin β bound to IBBs reveal that the protein can extend its solenoid structure by up to 10%, repositioning the C-terminal α-helix of different IBBs relative to the HEATs 11–19.

### Seven FG repeats bind importin β outer surface

**Figure 3.**
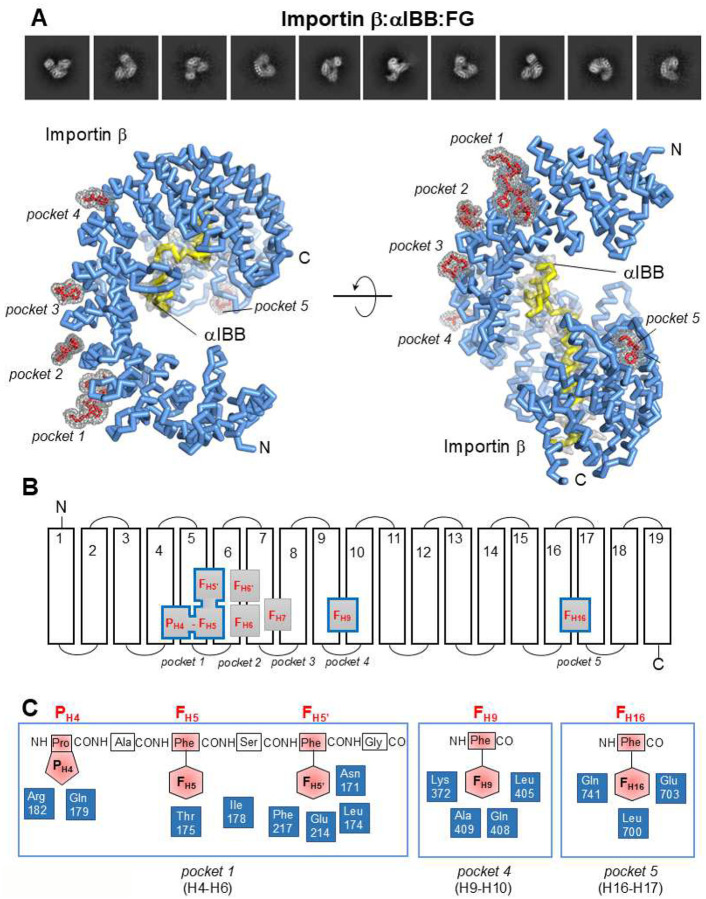
Cryo-EM structure of importin β:αIBB bound to FG-repeats. (**A**) Representative 2D class averages (top) and 3D structure of the importin β:αIBB:FG complex (bottom). Importin β is colored blue, the αIBB is yellow, and FGs are red. The final 3.3 Å cryo-EM density (gray) is overlaid onto the refined model for FG-peptides in chicken-wire style, and around the αIBB as a solid surface. (**B**) Schematic diagram of the FG-binding pockets on the outer surface of importin β. FH5 and FH5’ create a bipartite binding site. (**C**) Schematic diagram of the importin β residues (colored blue) that surround FGs (colored red) in pocket 1 (left), pocket 4 (center), and pocket 5 (right).

We reconstructed the structure of importin β:αIBB bound to the FG-rich nucleoporin Nsp1 at a maximum resolution of 3.2 Å (**Supplementary Fig. 1C**, Table 1). The reconstruction revealed strong peaks of density on the convex surface of importin β (Fig. 3A). We identified five major FG-binding pockets on the convex surface of importin β, formed at the interface between HEAT repeats H4-H6 (pocket 1), H6-H7 (pocket 2), H7-H8 (pocket 3), H9-H10 (pocket 4), and H16-H17 (pocket 5) (Fig. 3B). Pocket 1 binds the PA**F**S**F**G motif, positioning the two phenylalanine side chains (F_H5_ and F_H5’_) within a bipartite FG-binding pocket between H5 and H6. This pocket is formed by L174, I178, N171, and F217 (Fig. 3C), and represents the previously identified major FG-binding site^39^. A proline residue N-terminal to the FSFG motif (P_H4_) contacts the interface between H4 and H5. Pocket 2 is also bipartite, and is filled by an AFSF motif, which includes phenylalanine F_H6_ and F_H6’_ (Fig. 3B). In contrast, all other pockets are filled with a small density (Fig. 3A) consistent with a single phenylalanine: F_H7_ for pocket 3 (Fig. 3B), and F_H9_ and F_H16_ for pockets 4 and 5, respectively (Fig. 3C). Like pocket 1, the two smaller pockets 4 and 5 exhibit both hydrophobic and polar residues that interact with the phenyl ring and mainchain atoms (Fig. 3C). For example, F_H16_ contacts one hydrophobic residue, L700, one uncharged polar residue, Q741, and one negatively charged glutamate, E703. The side chain oxygen of the glutamate forms a hydrogen bond with the glycine main chains and has a non-bonded contact with the F_H16_ side chain (Fig. 3C). Thus, the chemistry of FG-binding to importin β includes both hydrophobic and polar contacts, suggesting FG-binding pockets are hydrated when importin β is not associated with FG-nups.

The cryo-EM reconstruction presented here identified all major FG-binding major sites (F_H5_ and F_H6_) described crystallographically^39^ and probed biochemically (F_H16_)^53,54^. Additionally, we experimentally validated five of the eight FG sites identified *in silico* using molecular dynamics simulations^38^, all located on the convex surface of importin β. Importantly, we found that the conformation of importin β observed in our cryo-EM reconstruction bound to FGs is similar to the importin:αIBB complex (**Supplementary Fig. 2A**), with an RMSD of approximately 1.9 Å. This indicates that FG-binding does not significantly alter the protein solenoid, at least in the αIBB-bound conformation^55^.

### Ran-GTP closes importin β solenoid

**Figure 4.**
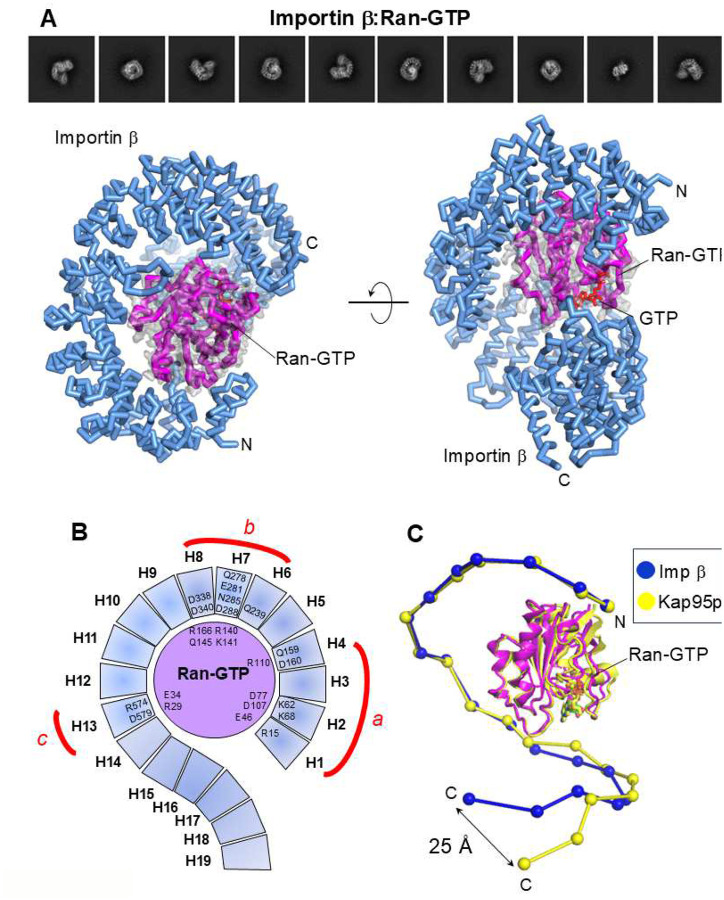
Cryo-EM structure of the importin β:Ran-GTP complex. (**A**) Representative 2D class averages (top) and the 3D structure of the importin β:Ran-GTP complex (bottom). Importin β is colored blue, while Ran-GTP is colored magenta. The final 3.2 Å cryo-EM density (gray) for Ran-GTP is overlaid with the refined model. (**B**) A schematic diagram illustrates the contacts between importin β and Ran-GTP within a distance of 4.5 Å. (**C**) The superimposition of Kap95p:Ran-GTP (PDB: 2BKU) with the importin β:Ran-GTP solved in this study is represented as beads-on-a-string. Each bead represents a HEAT repeat, while Ran-GTP is depicted in ribbon representation. Kap95p is colored yellow, and importin β is colored blue.

We elucidated the cryo-EM structure of the importin β:Ran-GTP complex at 3.3 Å resolution (**Supplementary Fig. 2D,** Table 1). In this structure (Fig. 4A), importin β is significantly closed around Ran-GTP, making extensive contact with HEATs 1–13. Specifically, Ran-GTP engages at three contact points with the concave surface of importin β, at HEATs 1–4 (contact point a), HEATs 6–8 (contact point b), and HEAT 13 (contact point c) (Fig. 4B). Ala178 is the last residue of Ran-GTP with discernable density in our reconstruction, whereas C-terminal residues 179–216 are disordered. The association between importin β and Ran-GTP comprises 10 salt bridges and 11 hydrogen bonds, burying 2,388 Å^2^ of the importin β concave surface. Perhaps the most striking finding is that the cryo-EM structure of the human importin β:Ran-GTP complex differs dramatically from the crystal structure of the Kap95p:Ran-GTP complex (PDB: 2BKU) with a staggering RMSD of 7.4 Å (Fig. 4C). In the cryo-EM structure, importin β C-terminus is completely closed relative to Ran-GTP, which is precisely the opposite of what is seen in the Kap95p:Ran-GTP complex. In vitreous ice, importin β N- and C-termini face each other with a maximum displacement of 19.4 Å compared to 40.4 Å in the Kap95p:Ran-GTP complex. Superimposing the cryo-EM structure of importin β with the Kap95p X-ray model reveals that the former has open, relaxed, and untwisted C-terminal HEATs 17–19 with a displacement of approximately 25 Å relative to the equivalent HEAT repeats in the yeast homolog (Fig. 4C). Thus, the previous crystal structure of Kap95p bound to Ran-GTP adopts a tertiary structure that is drastically different from that of human importin β.

### RanBP1 intercalates between importin β and Ran-GTP

**Figure 5.**
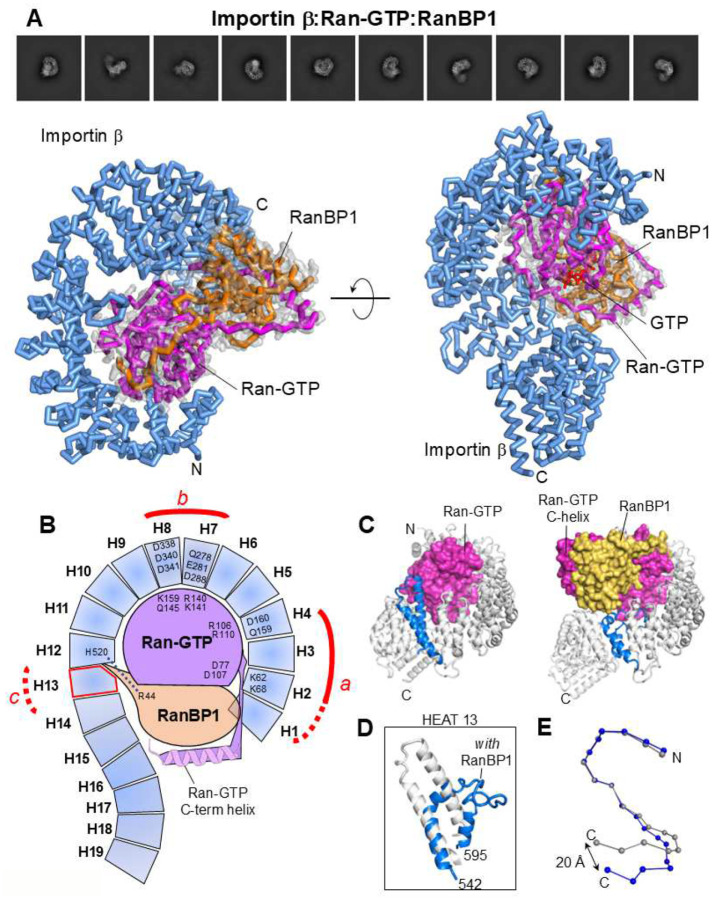
Cryo-EM structure of the importin β:Ran-GTP:RanBP1 complex. (**A**) Representative 2D class averages (top) and 3D structure of the importin β:Ran-GTP:RanBP1 complex (bottom). Importin β is colored blue, Ran-GTP is magenta, and RanBP1 is colored orange. The final 2.6 Å cryo-EM density (gray) of Ran-GTP:RanBP1 is overlaid onto the refined model. (**B**) Schematic diagram of importin β contacts made with Ran-GTP:RanBP1 within a 4.5 Å distance. (**C**) Ribbon diagrams of importin β:Ran-GTP (left) and importin β:Ran-GTP:RanBP1 (right) visualized. Importin β is shown in light gray with HEAT 13 colored blue, while Ran-GTP and RanBP1 are represented as solvent surfaces colored magenta and orange, respectively. (**D**) An overlay of HEAT 13 residues 542–595 from the structure of importin β:Ran-GTP (gray) and importin β:Ran-GTP:RanBP1 (blue) reveals a dramatic structural change caused by the association with RanBP1. (**E**) The superimposition of importin β in the conformation bound to Ran-GTP:RanBP1 (blue) and Ran-GTP (gray) is shown as beads-on-a-string. Ran-GTP and RanBP1 are not displayed.

Importin β and RanBP1 do not bind to each other without Ran, but the three proteins form a stable complex together^30,50,51^, which we reconstructed using cryo-EM SPA at 2.6 Å resolution (**Supplementary Fig. 1E**, Table 1). Unexpectedly, we found RanBP1 inserts itself at the interface between importin β and Ran-GTP, predominantly binding to the outer surface of Ran-GTP. The GTPase C-terminal moiety (res. 191–216) wraps around the RanBP1 core (Figs. 5A, B), recapitulating the tight embrace observed in the Ran-GTP:RanBP1 crystal structure^29^. Notably, RanBP1 makes limited contact with importin β in the ternary complex, limited to just one non-bonded interaction between R44 and H520 (Fig. 5B). Nonetheless, the association of RanBP1 with Ran-GTP profoundly influences the importin β solenoid, causing it to relax and extend, adopting a conformation that is even more elongated than when bound to xIBB (Fig. 2A).

RanBP1 alters the association of importin β with Ran-GTP in three significant ways. First, the C-terminal helix of Ran-GTP, disordered in the importin β:Ran-GTP complex (Fig. 4A), becomes structured with RanBP1, disrupting a salt bridge between Ran-GTP R15 and importin β E46 in HEAT 1 (Fig. 5B, contact point a). Second, the new binding interface between RanBP1 and Ran-GTP weakens the importin β:Ran-GTP binding interface at contact point c (red dashed line in Fig. 5B), resulting in the loss of two salt bridges and three hydrogen bonds. This is offset by 10 non-bonded interactions between RanBP1 R44 and HEAT 12 H520 (blue dashed lines in Fig. 5B). Third, the importin β:Ran-GTP association at HEAT 13 is partially disrupted due to a dramatic structural rearrangement of HEAT 13 (Fig. 5C). To accommodate both RanBP1 and Ran-GTP, HEAT 13 residues 542–595 bend by 90 degrees in the ternary complex (Fig. 5D), forming intramolecular contacts with the concave surface of importin β.

Thus, RanBP1’s association with importin β:Ran-GTP weakens two of the three bonding regions between the two proteins, specifically, points a and c, while keeping the bonding contacts between HEATs 2–8 seemingly unchanged (Fig. 5B). In the presence of RanBP1, the interaction between Ran-GTP and importin β decreases to six salt bridges and seven hydrogen bonds, with a total burial of 2,038 Å2 of importin β’s concave surface. Notably, the entire C-terminal cluster, consisting of HEATs 14–19, swings away from Ran-GTP in the presence of RanBP1, relaxing the solenoid in a way that contrasts significantly with the closed, snail-like conformation observed in the complex with Ran-GTP alone (Fig. 5E).

### Analysis of importin β domain motion

**Figure 6.**
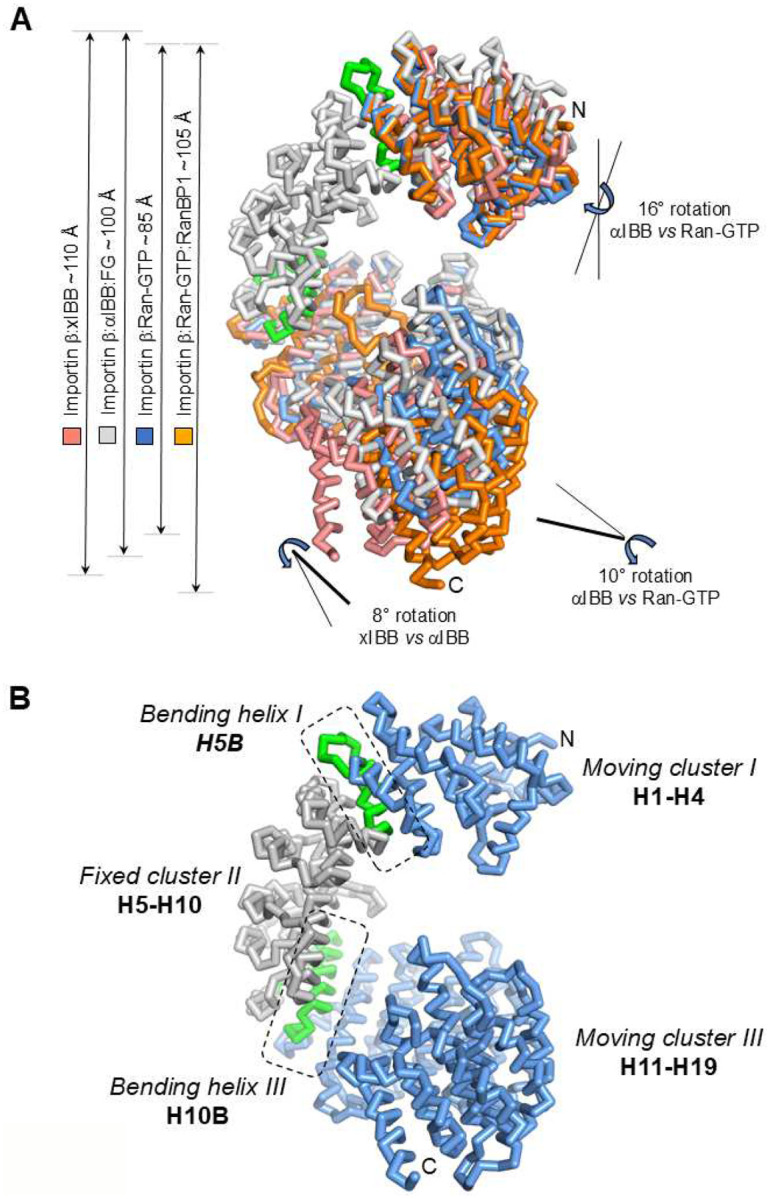
The curvature of importin β is influenced by its binding partners. (**A**) An overlay of all the importin β structures solved by cryo-EM in this study. Color coding: salmon, for importin β:xIBB; gray, for importin β:αIBB:FG; blue, for importin β:Ran-GTP; and orange, for importin β:Ran-GTP:RanBP1. Curved arrows and sticks indicate the angles of domain rotations. Specifically, HEATs 1–4 rotate 16 degrees clockwise, while HEATs 11–19 rotate 10 degrees counterclockwise when Ran-GTP binds to importin β relative to the αIBB-bound conformation. Similarly, there is an 8-degree counterclockwise rotation at HEATs 11–19 when importin β binds to the xIBB relative to αIBB. No distinct domain movement occurs in importin β bound to Ran-GTP:RanBP1 compared to Ran-GTP alone. (**B**) Rationalization of importin β domain movement based on DynDom analysis. Importin β consists of three clusters of HEAT repeats. Moving clusters I (HEATs 1–4) and III (HEATs 11–19) are depicted in blue, while the fixed cluster II (HEATs 5–10) is shown in gray. Bending helices H5B and H10B are shown in green.

To rationalize the range of importin β conformational dynamics observed in our cryo-EM reconstructions, we performed a global superimposition of the five cryo-EM structures of importin β determined in this study (Fig. 6A). Since all structures were obtained in a frozen-hydrated state, under physiological buffer, and refined at comparable resolution, it is reasonable to assume that the observed changes in importin β structure accurately reflect variations occurring during the nuclear import reaction. The first striking observation is the extent to which the importin β solenoid can stretch in response to binding effectors, ranging from approximately 85 to 110 Å, which is about one-quarter of the protein’s length. While the xIBB stabilizes the most extended conformation (around 110 Å), Ran-GTP instead strains the importin β solenoid, resulting in a globular assembly of roughly 85 Å (Fig. 6A). However, different binding effectors not only stretch the importin β solenoid but also induce local conformational changes at the N- and C-termini. The global RMSD among the four states described in this paper is significant, particularly for the Ran-GTP:RanBP1-bound complexes, namely, ~4.4 Å (xIBB vs. αIBB), ~7.3 Å (xIBB vs. Ran-GTP), ~9.3 Å (xIBB vs. Ran-GTP:RanBP1), ~3.6 Å (αIBB vs. Ran-GTP), ~8.2 Å (αIBB vs. Ran-GTP:RanBP1) and 7.2 Å (Ran-GTP vs. RanBP1). However, the changes are distributed anisotropically across different parts of the solenoid. This prompted us to analyze local domain movements using DynDom^56^.

We compared IBBs and found that the xIBB-bound importin β structure is ~10 Å more open than the αIBB structure due to motion in the C-terminal HEATs 11–19, which rotates by about 8 degrees in the xIBB complex relative to αIBB (Fig. 6A). More surprising is the role of Ran-GTP. Instead of inducing global domain motion, such as the opening of the C-terminal arch, as previously reported^41^, Ran-GTP affects the local conformation of importin β HEAT repeats 1–4 and 11–19, which close toward each other and slightly rotate clockwise (Fig. 6A). As a result of this movement, the maximum length of importin β decreases by ~15 Å and ~25 Å relative to the αIBB and xIBB, respectively (Fig. 6A), highlighting significant closure of the protein. However, comparing importin β bound to Ran-GTP with the Ran-GTP:RanBP1, DynDom did not detect any domain movement in terms of rotation of one domain relative to another. Importin β global stretching by approximately 20 Å occurs due to the insertion of RanBP1 between importin β and Ran-GTP, which is consistent with a rigid body movement.

Overall, DynDom analysis of importin β domain motion in response to binding effectors identified three clusters of HEAT repeats. Two moving clusters comprising N-terminal HEATs 1–4 (cluster I) and C-terminal HEATs 11–19 (cluster III) (blue in Fig. 6B) along with a fixed cluster II formed by HEATs 5–10 (gray in Fig. 6B), which is invariant in all our cryo-EM structures. Clusters I and III move relative to cluster II around the helices B of HEAT 5 (bending helix I) and HEAT 10 (bending helix III) (green in Fig. 6B). Ran-GTP binds to importin β, causing a 16-degree clockwise rotation in moving cluster I (H1-H4) and a 10-degree rotation in moving cluster III (H11-H19) while maintaining a fixed configuration in the region H5-H10. These rotations are linked to bending at the N- and C-termini around the helix B of H5 and H10, respectively (Fig. 6B). Because the N- and C-terminal clusters also twist around the two helices, the domain movement of clusters I and III is better described as bending rather than as hinge movement. The two movements of H1-H4 and H11-H19 must account for the known activities of Ran-GTP, namely, reducing FG-nup binding avidity and displacing cargo from importin β.

### Ran-GTP binding to importin β closes FG-binding pockets

**Figure 7.**
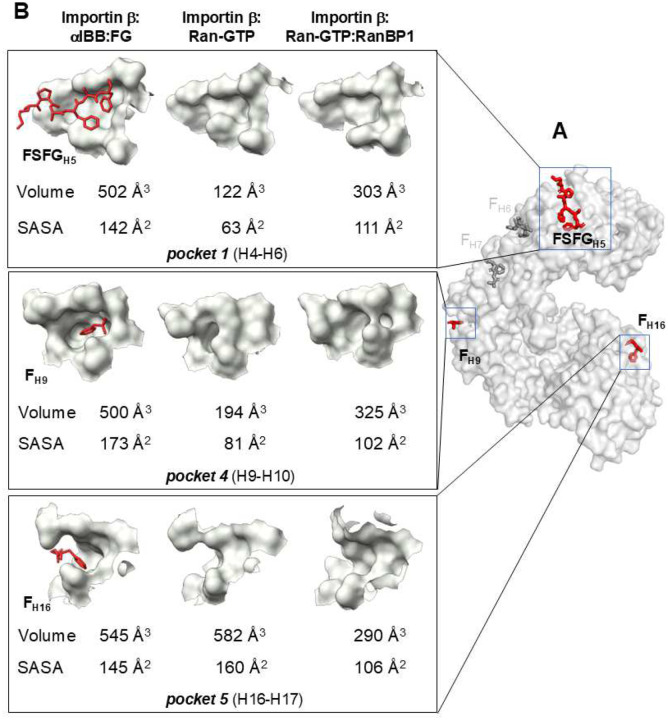
Ran-GTP allosterically closes FG-binding pockets. (**A**) Importin β is shown as a gray solvent surface, while the bound FG-peptides are depicted as red and gray sticks. (**B**) Magnified view of the FG-binding pockets 1 (top) and 4 (bottom) shown as a solvent surface from three importin β complexes: bound to αIBB (left), Ran-GTP (center), and Ran-GTP:RanBP1 (right). The FGs bound to the pockets are shown as red sticks. Pockets 1, and 4 notably shrink after binding to Ran-GTP and Ran-GTP:RanBP1. The volume and solvent-accessible surface area (SASA) of each pocket are calculated using FPocketWeb^57,58^, is indicated for each of the three conformations of importin β.

Ran-GTP reduces the affinity of importin β for FG-nups, yet none of the crystal structures determined thus far have explained how. To address this question, we focused on the outer surface of frozen-hydrated importin β, which was solved in complex with αIBB:FG-repeats (Fig. 3A), Ran-GTP (Fig. 4A), and Ran-GTP:RanBP1 (Fig. 5A). Guided by the atomic coordinates of FGs bound to importin β:αIBB (Fig. 3A), we measured the volume and solvent-accessible surface area (SASA) of each of the five pockets in importin β using FPocketWeb^57,58^. We found that the five pockets have volumes ranging from 502 to 154 Å3, displaying characteristics that lie between pocket-like invaginations and cavities (**Supplementary Table 1**). We then compared the volume and shape of these FG-binding pockets in importin β bound to Ran-GTP and Ran-GTP:RanBP1. Strikingly, we observed a substantial size reduction in cavity volume and SASA in two pockets: pocket 1 (H4-H6) and pocket 4 (H9-H10) (Fig. 7A, B). Notably, the bipartite pocket between H5-H6 displayed a significant decrease in solvent-accessible volume at the first site (H5-H6) (Fig. 7B), diminishing from 502 Å^3^ to 122.0 Å^3^ and 303 Å^3^, respectively. This site is likely vital as a single point mutation at I178D abolishes importin β nuclear import in permeabilized cells^39^. Similarly, the pocket between H9-H10 (pocket 4) that binds F_H9_ exhibited a noteworthy reduction in solvent-accessible volume, with the F_H9_ pocket decreasing from 500 Å^3^ to 194 Å^3^ and 325 Å^3^ (Fig. 7B). Notably, F_H9_ binds at the bending region identified when Ran-GTP is associated with importin β (green in Fig. 6B). Finally, pocket 5 located between H16-H17 at the C-terminal end undergoes a remarkable decrease in solvent-accessible volume only in the importin β:Ran-GTP:RanBP1 complex, diminishing from 545 Å^3^ to 290 Å^3^ (Fig. 7B). In contrast, Ran-GTP and RanBP1 binding to importin β did not perturb pockets 2 and 3, which are very shallow (Fig. 3C).

**Figure 8.**
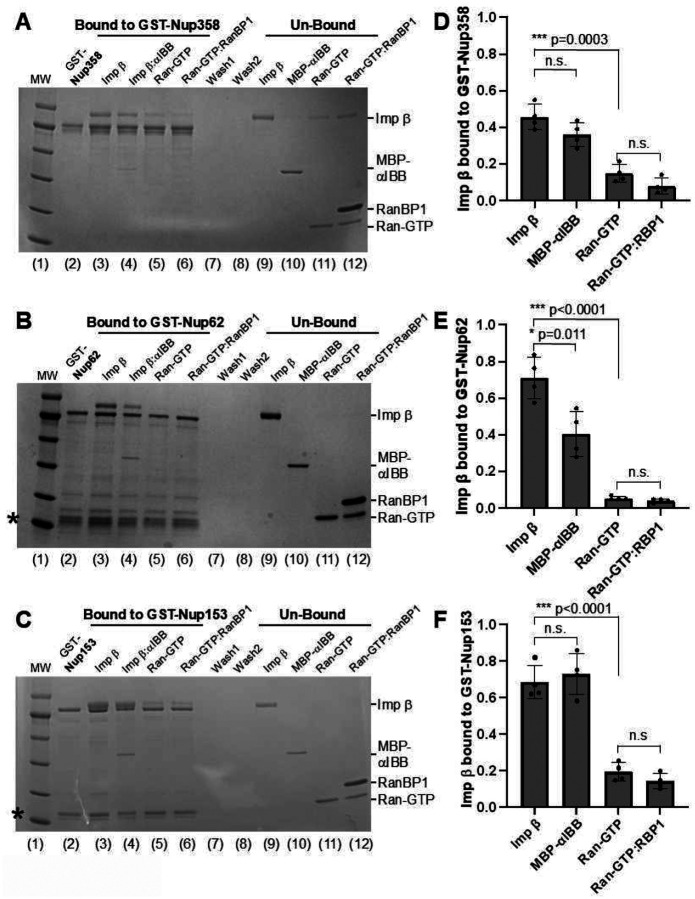
Pull-down assay of importin β and FG-nups. Representative SDS-PAGE gels of the pulldown experiment in which (**A**) GST-Nup358 (res. 2503–2893), (**B**) GST-Nup62, and (**C**) GST-Nup153 (res. 946–1472) were coupled to glutathione agarose beads and incubated with equal amounts of purified importin β (lane 3), importin β:αIBB (lane 4), importin β plus Ran-GTP (lane 5) and importin β plus Ran-GTP:RanBP1 (lane 6). Washes are in lanes 7–8, and unbound fractions are in lanes 9–12. M.W. = molecular weight markers. Free GST co-purified with GST-FG-nups is visible at the bottom of the gel, indicated by an asterisk (*). (**D-F**) Quantification of importin β bands bound to GST-FG-nups from replicate SDS-PAGE gels (n=4). Band intensity was measured using ImageJ^70^. Histogram columns represent the ratio of importin β band intensity (black circles) normalized by the intensity of the GST-FG-nup band, with relative standard deviation indicated by error bars. A significant (p<0.01) deviation from the importin β:GST-FG-nup ratio was assessed using a two-tailed Student’s t-test, assuming unequal variance. In this analysis, *p<0.05 and ***p<0.001 are considered statistically significant, while n.s. indicates not significant.

We experimentally validated the prediction that importin β bound to Ran-GTP:RanBP1 has diminished binding affinity for FG-nups, like in the Ran-GTP-bound state. We immobilized GST-tagged Nup358, Nup62, and Nup153 on glutathione beads, which are representative of FG-nups localized to the NPC cytoplasmic face, internal channel, and nuclear basket, respectively (Fig. 8A–C, lane 2). Using a pulldown assay, we found all FG-nups efficiently pulled down free importin β and importin β:αIBB (Fig. 8A–C, lanes 3–4, quantified in Fig. 8D–F). Nup62 bound free importin slightly better than in complex with αIBB (Fig. 8B, E), corroborating a previous report^34^. At the same time, no statistically significant difference was observed for Nup358 and Nup153 (Fig. 8A, C lanes 3–4, quantified in Fig. 8D, F). In the presence of Ran-GTP alone or Ran-GTP:RanBP1, importin β exhibited dramatically reduced binding to all tested FG-nups (Fig. 8A–C, lanes 5–6, quantified in Fig. 8D–F). Ran-GTP and Ran-GTP:RanBP1 dissociated ~90–95% of all importin β from GST-Nup62 and -Nup358 (Fig. 8D, E), whereas about 20–25% of importin β remained bound to GST-Nup153 (Fig. 8F). No statistically significant difference was observed between Ran-GTP alone and Ran-GTP-RanBP1, both of which equally displaced importin β from FG-nups (Fig. 8D–F). Therefore, Ran-GTP binding to the concave surface of importin β induces a long-distance conformational change, which reduces the solvent-accessible surface of at least two FG-binding pockets on the outer surface, thereby disrupting FG association *in vitro*. The ternary importin β:Ran-GTP:RanBP1 complex also exhibits a low affinity for FG-nups due to the closure of three FG-binding pockets 1, 4, and 5 (Fig. 7B).

### IBB and Ran-GTP bind to partially different regions of importin β

**Figure 9.**
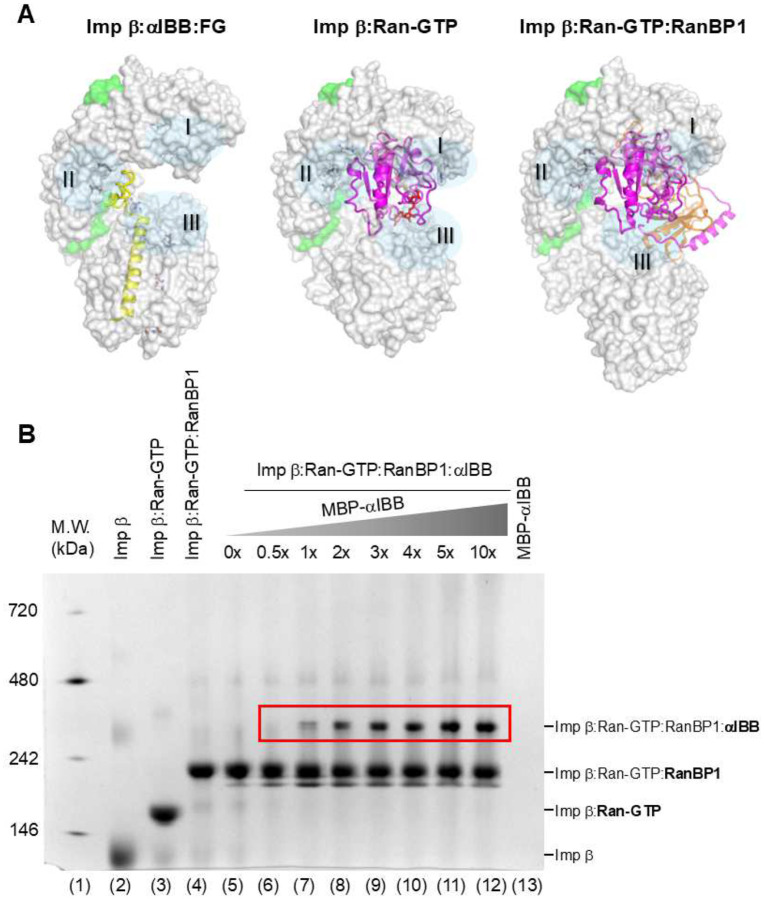
Importin β binds both Ran-GTP and αIBB in the presence of RanBP1. (**A**) Solvent surface representations of importin β bound to αIBB (yellow ribbon, left), Ran-GTP alone (magenta, center), and Ran-GTP:RanBP1 (magenta and orange, right). Semitransparent blue circles labeled I, II, and III highlight three binding regions in importin β. (**B**) Native gel electrophoresis shows a sequential addition of Ran-GTP (lane 3) and Ran-GTP:RanBP1 (lane 4) to purified importin β (lane 2) along with a titration of αIBB (lanes 5–12) against a preformed importin β:Ran-GTP:RanBP1 complex. Lane 1 has molecular weight (M.W.) native gel markers. The MBP-αIBB control is in lane 13, although this fusion protein is basic and does not migrate on a native acrylamide gel.

We next investigated the importin β binding interface for IBB and Ran-GTP and identified three binding regions: HEATs 1–6 (region I), HEATs 7–13 (region II), and HEATs 14–19 (region III) (Fig. 9A). These three regions are similar but not identical to the three moving clusters identified through local domain motion analysis (Fig. 6B). Both αIBB and xIBB domains do not contact region I, form only two salt bridges with region II, and create over ten hydrogen bonds with importin β C-terminal HEATs 14–19, which is part of region III (**Supplementary Fig. 3A**). In contrast, Ran-GTP primarily binds to importin β through the N-terminal regions I and II (**Supplementary Fig. 3B**). The interaction with importin β involves five hydrogen bonds in region I and five salt bridges along with six hydrogen bonds in region II. Residues E281 and D288 in region II are the only residues in importin β bound by either IBB or Ran-GTP, both engaging in electrostatic interactions (**Supplementary Fig. 3B**). Thus, the binding sites for IBBs and Ran-GTP in importin β are mainly distinct. IBB associates primarily with the C-terminal region III (HEATs 7–19), whereas Ran-GTP binds to the N-terminal arch comprising HEATs 1–13, validating previous mutagenesis studies^17^, with some overlap in HEAT 13. Strikingly, in the presence of Ran-GTP:RanBP1 (**Supplementary Fig. 3B**), three salt bridges and three hydrogen bonds between Ran-GTP and importin β in region II are disrupted by RanBP1, resulting in an overall opening of HEATs 14–19, potentially creating space to accommodate the IBB.

We then asked whether RanBP1 would facilitate the formation of a larger assembly comprising both αIBB and Ran-GTP. We titrated an equimolar quantity of purified Ran-GTP against 5 μg of importin β and visualized the relative association using native PAGE (Fig. 9B, lanes 2–3). We assembled the importin β:Ran-GTP:RanBP1 complex by adding a stoichiometric amount of RanBP1 (Fig. 9B, lane 4), resulting in a ternary complex that shifted upward compared to importin β:Ran-GTP (Fig. 9B, lane 3). Finally, we titrated a 0–10-fold excess of αIBB fused to the maltose binding protein (MBP-αIBB) against the importin β:Ran-GTP:RanBP1 ternary complex (Fig. 9B, lanes 5–12). Although the MBP-αIBB chimera is basic and does not migrate on a native gel (Fig. 9B, lane 13), it significantly shifted the migration of the trimeric importin β:Ran-GTP:RanBP1 complex. In the presence of MBP-αIBB, a new band shifted upwards was observed, corresponding to a tetrameric complex formed by importin β:Ran-GTP:RanBP1:MBP-αIBB (Fig. 9B, lanes 7–12). Thus, the association of RanBP1 with importin β and Ran-GTP promotes binding to αIBB, which is a proxy for the classical import complex (e.g., importin α1:NLS-cargo).

## DISCUSSION

This study aimed to elucidate two key mechanisms of nuclear import that have remained unclear despite decades of extensive research. On one hand, we sought to determine how Ran-GTP dissociates importin β from high-affinity FG-binding sites lining the NPC, allowing import complexes to move through the selective barrier instead of clogging the NPC^17^. On the other hand, we asked how Ran-GTP disassembles the import complex, releasing the cargo into the nucleus and preventing futile rounds of stochastic, Ran-GTP-induced dissociation and reassociation within the NPC^59^. To address these questions, we determined five unbiased structures of importin β bound to various effectors encountered in a typical import reaction and complemented the structural data with biochemical measurements of importin β activity. Overall, we shed light on three aspects of importin β biology relevant to answering the two questions above.

First, we present a comprehensive description of the importin β solenoid conformational landscape through a comparative analysis of the structure of importin β obtained in vitreous ice. Our analysis suggests that importin β resembles an intrinsically disordered protein, in which the nineteen HEAT repeats maintain their secondary structures but lack a tertiary structure, as the solenoid fluctuates among various conformations, sampling different states that are possibly separated by low energy barriers^46^. We provide evidence that distinct importin β conformers bind to, and are stabilized by αIBB, xIBB, Ran-GTP, and Ran-GTP:RanBP1, resulting in structurally distinct solenoids. We identified three HEAT repeat clusters in importin β, two of which (HEATs 1–4 and 11–19) move relative to an invariant core (HEATs 5–10) by bending around helices H5B and H10B (Fig. 6B). This parameterization of importin β conformational dynamics could not have been achieved using X-ray crystallography. All previously determined crystal structures of importin β exhibit significant fluctuations primarily due to the type of crystallization agents, pH, and lattice forces, unlike cryo-EM structures obtained under quasi-physiological conditions. We provide the first experimental visualization of five FG-binding pockets on the outer surface of importin β. At the current resolution (~3.2 Å), we did not observe significant conformational changes in the importin β solenoid upon FG-binding, suggesting that FG-repeats function like molecular Velcro^60^, decorating the outside of importin β without inducing substantial fluctuations in the import receptor.

Second, we analyzed how Ran-GTP binds to importin β, regulating its activities essential for nuclear transport. The GTPase stabilizes a highly restrained conformation of the importin β solenoid that wraps around Ran-GTP by making three contact points (Fig. 4B). This tripartite recognition forces importin β to adopt a globular conformation that alters the protein curvature, closing two of the five FG-binding pockets on its outer surface. Consequently, the association of Ran-GTP with the concave surface of importin β triggers an allosteric change on the convex surface, affecting both the volume and the solvent-accessible surface of the FG pockets. By doing this, Ran-GTP reduces the affinity for FG-nups lining the NPC but does not eliminate it entirely, enabling the import complex to continue moving through the NPC and bypassing the permeability barrier. Remarkably, Ran-GTP does not open the C-terminus of importin β, as previously reported. The cryo-EM structure of human importin β bound to Ran-GTP presented in this study significantly differs from a previous crystal structure of yeast Kap95p bound to Ran-GTP^41^, demonstrating that Kap95p is structurally unrepresentative of human importin β. As a corollary, the proposed model for Ran-GTP-mediated cargo displacement based on a change in the helicoidal pitch of the importin β C-terminus is unlikely. Our cryo-EM reconstructions suggest that Ran-GTP and IBBs partially overlap in their binding to importin β region II (Fig. 9A), where the two binding effectors compete for a limited number of residues. The IBB domain primarily associates with the C-terminal region III (HEATs 7–19), while Ran-GTP binds to HEATs 1–13 at three contact points (Fig. 4B). We envision a sequential mechanism for cargo release that begins with the association of Ran-GTP with importin β at contact point a (Fig. 4B). This interaction causes a 16-degree clockwise rotation of the moving cluster I (HEATs 1–4) relative to the fixed cluster II (HEATs 5–10) and a 10-degree counterclockwise rotation of cluster III (H11-H19) (Fig. 6B). The globularization of importin β around Ran-GTP reduced the N-to-C distance by ~15 Å relative to the αIBB-bound conformation, breaking the bonds between αIBB and HEATs 13–19 in region III (**Supplementary Fig. 3A**). Thus, Ran-GTP allosterically displaces the import complex, triggering long-range conformational changes in the importin β solenoid that displace the IBB domain.

Third, our study provides a molecular explanation of the role of RanBP1 in the import reaction. RanBP1 promotes the translocation of the classical nuclear import complex through the NPC^61,62^, a function also conserved in yeast^63^. Previous studies found that RanBP1 increases the affinity of both Ran-GDP and Ran-GTP for importin β to the same level, suggesting that the nucleotide-bound state of Ran does not affect the stability of the importin β:Ran:RanBP1 complex^50^. In the literature, RanBP1 is primarily implicated in the disassembly of the import complex^30^, serving as the key player that removes importin β’s inhibition of GTP hydrolysis, directly promoting the dissociation of the import complex at Nup358, where RanGAP1 is crosslinked through sumoylation. Interestingly, this process also requires the IBB domain of importin α *in vitro*^30^. Our study finds that RanBP1 triggers dramatic structural and functional changes in the importin β:Ran-GTP complex. In the presence of Ran-GTP and RanBP1, importin β retains a low affinity for FG-nups and can hold on to the cargo due to an extended position of the C-terminal HEATs 11–19. Thus, we hypothesize that a transient assembly of the import complex (importin β:α1:NLS-cargo) bound to both Ran-GTP and RanBP1 may exhibit sufficient stability and decreased avidity for FG-nups, allowing movement across the NPC. This would enable the import complex to traverse the permeability barrier and pass through the central channel as a complete entity, circumventing unproductive cycles of cargo release and reassociation. This transient assembly would possess the properties of importin β bound to Ran-GTP in terms of affinity for FGs, yet it would remain attached to the NLS-cargo like an import complex formed in the cytoplasm. The final disassembly of the import complex would take place at the NPC basket, where import complexes encounter a higher concentration of nuclear Ran-GTP.

In summary, we have deciphered the complete conformational dynamics of importin β and provided insight into the function of Ran-GTP. This GTPase has evolved to modulate the affinity of β-karyopherins for FG-nups by allosterically changing the curvature and, therefore, the volume and solvent-accessible surface of FG-binding pockets. We propose that the structural principles elucidated in this work for importin β are conserved and applicable to members of the β-karyopherin family involved in nuclear import and export. It is possible that β-karyopherins and Ran-GTP coevolved under selective pressure to achieve efficient and rapid nuclear import.

## METHODS

### Cloning, expression, and purification of recombinant proteins

The importin β:α1 heterodimer was cloned in a pACYCDuet-1 and expressed in BL21-DE3 *E. coli* cells. Protein expression was induced with 500 μM IPTG for 3 hours at 30 °C, and the heterodimer was purified to homogeneity, as previously described^64,65^. Untagged importin β was separated from the importin β:α1 complex bound to the nickel agarose beads (GenScript) using a Separation Buffer (20 mM Tris-HCL, pH 8.0, 150 mM NaCl, 250 mM MgCl2 and 3 mM 2-mercaptoethanol). Importin β was further purified by size-exclusion chromatography (SEC) on a Superdex 200 26/600 preparative column equilibrated in β SEC Buffer (20 mM Tris-HCL, pH 8.0, 150 mM NaCl, 1 mM PMSF, and 3 mM 2-mercaptoethanol).

The importin α1 IBB domain N-terminally fused to the Maltose Binding Protein (MBP-αIBB) was expressed in BL21-DE3 *E. coli* cells. Protein expression was induced with 500 μM IPTG for 3 hours at 30 °C. Cells were resuspended in IBB Lysis Buffer (20 mM Tris-HCL, pH 8.0, 200 mM NaCl, 1 mM PMSF, 3 mM 2-mercaptoethanol, 1mM EDTA, and 0.1% Triton X, DNase, and RNase) and after sonication, the soluble fraction was bound to amylose resin (New England Biolabs). The protein was washed using a lysis buffer and then eluted with a lysis buffer with 15 mM maltose. MBP-αIBB was further purified by SEC using a Superdex 200 16/600 preparative column equilibrated with IBB SEC Buffer (20 mM Tris-HCL, pH 8.0, 200 mM NaCl).

Ran-GTP was expressed and purified using a Ran-Q69L mutant that remains in a GTP-bound conformation. Ran-GTP cloned in a pET28a-PPase construct was expressed in BL21-DE3 *E. coli* expression strain supplemented with kanamycin^34^. Protein was expressed at 18 °C overnight with 500 μM IPTG. Cells were resuspended in Ran Lysis Buffer (50 mM KPO4 pH 7.0, 250 mM NaCl, 2 mM MgCl2, 1 mM PMSF, and 3 mM 2-mercaptoethanol) and after sonication, the soluble fraction was bound to low-density nickel agarose beads (GoldBio). The protein was washed using a lysis buffer containing 10 mM imidazole and then eluted with a lysis buffer with Ran Elution Buffer (50 mM KPO4 pH 7.0, 250 mM NaCl, 2 mM MgCl2, 1 mM PMSF, and 3 mM 2-mercaptoethanol, 150 mM imidazole). Ran-GTP was further purified by SEC using a Superdex 200 16/600 preparative column equilibrated with Ran SEC Buffer (50 mM KPO4 pH 7.0, 250 mM NaCl, 2 mM MgCl2, 1 mM PMSF, and 3 mM 2-mercaptoethanol). The importin β:Ran-GTP complex was formed by incubating importin β and Ran-GTP at 1 to 4 molar ratio at 4°C for 30 minutes.

RanBP1 was cloned in a pET28a-PPase vector and expressed in BL21-DE3 *E. coli* cells^34^. Protein expression was induced with 500 μM IPTG for 3 hours at 30 °C. Cells were resuspended in a Low Salt Lysis Buffer (20 mM Tris-HCL, pH 8.0, 150 mM NaCl, 1 mM PMSF, and 3 mM 2-mercaptoethanol), lysed by sonication, and the soluble fraction isolated by centrifugation and incubated with low-density nickel agarose beads (GoldBio). The protein was washed using a Low Salt Lysis Buffer containing 5–20 mM imidazole, 50 mL for each wash, then eluted with Elution Buffer (20 mM Tris-HCL, pH 8.0, 150 mM NaCl, 1 mM PMSF, and 3 mM 2-mercaptoethanol, 150 mM imidazole). RanBP1 was further purified by SEC using a Superdex 200 16/600 preparative column equilibrated with PBS plus 0.1 mM PMSF.

The FxFG-rich fragment (residues 497–608) of yeast Nsp1^66^ was cloned into expression vector pMW172, expressed, and purified as described^67^. The protein was expressed for 3 hours at 30 °C after adding 400 μM IPTG to cells at an optical density at 600 nm of ~0.6. The Nsp1 fragment was purified in a High Salt Lysis Buffer (20 mM Tris-HCL, pH 8.0, 600 mM NaCl, 1 mM PMSF, and 3 mM 2-mercaptoethanol and 5 % glycerol) and bound to low-density nickel agarose beads (GoldBio). The protein was washed with a high salt buffer containing 5/10/20 mM imidazole, 10 mL for each wash, then eluted with Elution Buffer (20 mM Tris-HCL, pH 8.0, 150 mM NaCl, 1 mM PMSF, and 3 mM 2-mercaptoethanol, 150 mM imidazole) supplemented with 1.25 % glycerol. The importin β:α1:FG complex was formed by adding a 2-fold molar excess of Nsp1 (497–608) to purified importin α1/β and used immediately for vitrification.

GST-tagged Nup358 (res. 2503–2893)^68^, full-length Nup62^68^, and Nup153 (res. 946–1472)^68^ were cloned in vector pGEX-4T1 and expressed in BL21-DE3 *E. coli* cells. Protein expression was induced with 500 μM IPTG for 3 hours at 30 °C. Cells were resuspended in a Medium Salt Lysis Buffer (20 mM Tris-HCL, pH 8.0, 300 mM NaCl, 0.2% Triton X, 1 mM PMSF, and 3 mM 2-mercaptoethanol) plus protease inhibitor, and after sonication, the soluble fraction was bound to glutathione beads (GenScript). The protein was washed using a lysis buffer containing 0.2% Triton X and then buffer-exchanged to Protein Binding Buffer (20 mM HEPES, pH 7.0, 150 mM KOAc, 2 mM Mg(OAc)2, 1 mM dithiothreitol, and 0.1% Tween) plus protease inhibitors. The immobilized protein was used immediately to avoid degradation.

A peptide comprising XRIP1α residues 6–47 (xIBB) was synthesized by GenScript.

### Pulldown assays and native gel electrophoresis

GST-tagged fragments of Nup358, Nup62, and Nup153 were immobilized on glutathione beads (GoldBio) and resuspended in Pulldown Binding Buffer (20 mm HEPES, pH 7.0, 150 mm KOAc, 2 mm Mg(OAc)2, 1 mm dithiothreitol, and 0.1% Tween). Pulldown assays were carried out as described^34,69^. Briefly, 3 μM purified importin β was added to glutathione beads pre-coupled with GST-Nups and incubated for one hour at 4°C. Unbound fractions were washed away, and beads were washed three times with Pulldown Wash Buffer (20 mm HEPES, pH 7.0, 150 mm KOAc, 2 mm Mg(OAc)2, 1 mm dithiothreitol, and 0.1% Tween). At this point, different 500 μL batches of beads coupled with GST-Nups bound to importin β were incubated with 10 μM MBP-αIBB, or 10 μM Ran-GTP, or 10 μM Ran-GTP plus 30 μM RanBP1; incubated for another one hour at 4°C, then Pulldown Wash Buffer, and bound fractions analyzed on a 8–16% Tris-Glycine mini protein gel (Invitrogen, XP08162BOX). SDS-PAGE gels were run in triplicate (n=3), and the average ratio of importin β band intensity normalized to each GST-FG-nup through densitometry using ImageJ^70^. We employed a two-tailed Student’s t-test, assuming unequal variances, to determine statistical significance between the means of the control and experimental pulldowns. The alpha parameter had to be outside the 99^th^ percentile for statistical significance (p<0.01). Error bars indicate the standard deviation of the mean.

Native Gel electrophoresis was performed using NativePAGE^™^ Bis-Tris Mini Protein Gels, 3–12% (Invitrogen, BN1001). Briefly, 5 μg of importin β:α1 or importin β were incubated with 1x, 3.5x, 7x, 14x, and 21x molar ratios of Ran-GTP on ice for one hour. After one hour, 3.75 μL of 4x Loading buffer (Invitrogen, BN2003) was added to the sample to reach a final volume of 15 μL. Samples were then loaded onto a 3–12 % Bis-Tris native gel, run the gel with Bis-Tris non-denaturing running buffer (Invitrogen, BN2007) at 150 V for 90 minutes, at 4°C. The gel was stained with Coomassie Brilliant Blue-G-250 and destained overnight in 50% (v/v) methanol in water with 10% (v/v) acetic acid.

### Vitrification and data collection

For all importin β complexes studied in this paper, typically 2.0 μl of importin β pre-bound to αIBB, xIBB, αIBB:Nsp1, Ran-GTP or Ran-GTP:RanBP1 at 2 μg/μl final concentration were applied to a 300-mesh copper Quantifoil R 1.2/1.3 holey carbon grid (EMS) previously glow-discharged negatively for 60 sec at 15 mA using an easiGlow (PELCO). Only for the importin β:αIBB:Nsp1 complex (referred to herein as importin β:αIBB:FG) and importin β:Ran-GTP:RanBP1, we used 200-mesh gold Quantifoil R 1.2/1.3 grid (EMS) instead of standard carbon grids. All grids were blotted for 5 sec at blot force 5 and frozen in liquid ethane using Vitrobot Mark IV (FEI). All micrographs were pre-screened in-house on a 200 kV Glacios2 equipped with a Falcon 4i detector at the UAB Cryo-EM Facility. EPU software was used for data collection using the accurate positioning mode. All high-resolution datasets were collected at Titan Krios microscopes available at three federal facilities equipped with either a K3 direct electron detector camera (e.g., NCEF and NCCAT) or a Falcon 4i (e.g., S2C2 and NCCAT). 9,497 micrographs were collected for importin β:xIBB in super-resolution mode plus energy filter at 20 eV with an image pixel size of 0.436 Å at 105,000x magnification, a nominal total dose of 50 e/Å^2^, 40 frames, and defocus range −1.0 to −2.5 μm. 27,987 micrographs were collected for importin β:αIBB in super-resolution mode plus energy filter at 20 eV with an image pixel size of 0.371 Å at 165,000x magnification, a nominal total dose of 50 e/Å2, 40 frames, and defocus range −0.8 to −2.5 μm. 18,016 micrographs were collected for importin β:αIBB:FG plus energy filter at 20 eV with an image pixel size of 0.959 Å at 130,000x magnification, a nominal total dose of 50 e/Å^2^, 40 frames, and defocus range −0.8 to −2.5 μm. 13,468 micrographs were collected for importin β:Ran-GTP in super-resolution mode plus energy filter at 20 eV with an image pixel size of 0.436 Å at 105,000x magnification, a nominal total dose of 50 e/Å^2^, 40 frames, and defocus range −1.0 to −2.5 μm. 11,100 micrographs were collected for importin β:Ran-GTP:RanBP1 plus energy filter at 20 eV with an image pixel size of 0.371 Å at 165,000x magnification, a nominal total dose of 50 e/Å^2^, 40 frames, and defocus range −0.8 to −2.5 μm. All collection parameters are in Table 1.

### Cryo-EM single particle analysis (SPA)

Micrographs were subjected to patched motion-correction, patched CTF (Contrast Transfer Function) estimation using CTFFIND4^71^. All subsequent steps of SPA were carried out using cryoSPARC version 4.2.1^72^. Particle picking was done using one round of template picker and at least three rounds of Topaz training and extraction^73^, followed by extracting particles from micrographs. In total, we picked 4.3 million for importin β:xIBB, 11.9 million for importin β:αIBB, 5.8 million particles for importin β:αIBB:FG, 6.5 million for importin β:Ran-GTP, and 9.6 million for importin β:Ran-GTP:RanBP1. The initial maps were reconstructed by using a combination of selected 2D classes and *ab initio* reconstruction. In total, 293,677, 157,234, 593,354, 1,400,279, and 721,883 particles were used to calculate 3D densities for importin β:xIBB, importin β:αIBB, importin β:αIBB:FG, importin β:Ran-GTP, and importin β:Ran-GTP:RanBP1, respectively. The 3D maps were further refined with heterogeneous 3D classification, homogeneous refinement with CTF and defocus refinement options, and post-processing using a 10xGPU cluster. The final densities were sharpened using *phenix.auto_sharpen*^74^.

### Model building, refinement, and structure analysis

All atomic models were built using Coot^75^ and ChimeraX^76^ followed by several rounds of rigid-body, real-space, and B-factor refinement using *phenix.real_space_refinement*^77^. All final models were validated using MolProbity^78^ (Table 1). All images of models and cryo-EM maps were generated using ChimeraX^76^ and PyMol^79^. RMSD between superimposed PDBs, structural comparison, and domain motion analysis were done using the DynDom server^56^ and SuperPose 1.0^80^. FPocketWeb^57,58^ was used to identify and characterize binding pockets within importin β. Binding interfaces were analyzed using PISA^81^ and PDBsum^82^. The cartoon model was generated in part using BioRender.com, which is licensed at UAB.

## Figures and Tables

**Table 1.** CryoEM data collection, maps, and model refinement statistics

Data Parameter	Importin β: αIBB	Importin β: xIBB	Importin β: αIBB:FG-nup	Importin β: Ran-GTP	Importin β: Ran-GTP:RanBP1
Facility	S2C2	NCEF	NCCAT	NCCAT	S2C2
Scope	Krios 300 kV	Krios 300 kV	Krios 300 kV	Krios 300 kV	Krios 300 kV
Detector	Falcon 4i	Gatan K3	Falcon 4i	Gatan K3	Falcon 4i
C2 aperture (μm)	100	100	100	100	100
Cs	2.7	2.7	2.7	2.7	2.7
Nominal magnification	165,000x	105,000x	130,000x	105,000x	165,000x
No. micrographs	27,987	9,497	18,016	13,468	10,100
Pixel size (Å/px)	0.371	0.436	0.959	0.436	0.371
Spot Size	7	7	7	7	7
Exposure (sec)	2.98	2.98	2.98	2.98	2.98
Dose (e^−^/px/sec)	7	12.8	7	12.8	7
Total dose (e^−^/Å^2^)	50	50	50	50	50
No. Fractions	40	40	40	40	40
Defocus range (μm)	−0.8 to −2.5	−1.0 to −2.5	−0.8 to −2.5	−1.0 to −2.5	−0.8 to −2.5
Exposures per hole	1	1	1	1	1
**Model statistics**					
PDB / EMDB entry	9N86 / 49116	9N87 / 49117	9BFC / 44492	9BAW / 44412	9N85 / 49114
Particles per reconstruction	157,234	293,677	593,354	1,400,279	721,883
Resolution Map (Å) FSC (0 / 0.143 / 0.5)	3.0 / 3.3 / 3.7	3.0 / 3.4 / 4.0	3.1 / 3.2 / 3.6	3.2 / 3.3 / 3.6	2.5 / 2.6 / 2.8
Model-to-Map Correlation Coeff (CC)	0.86	0.82	0.81	0.83	0.87
Chains / Residues	2 / 919	2 / 921	7 / 946	3 / 1,046	
Bonds (RMSD) Length (Å) / Angles (°)	0.002(0) / 0.6 (0)	0.002(0) / 0.6(2)	0.004(0) / 0.8 (9)	0.003 (0) / 0.6 (0)	0.003 (0) / 0.6 (6)
MolProbity Score / Clash	1.9 / 6.3	2.4 / 10.8	1.4 / 1.7	1.9 / 5.3	2.2 / 6.1
Ramachandran (%) Out / Allow / Favorite	0.0 / 2.5 / 97.5	0.0 / 4.8 / 95.2	0.0 / 4.2 / 95.7	0.1 / 3.2 / 96.7	0.7 / 7.8 / 91.5
Rama-Z (RMSD) Whole / helix / loop	1.2 (0.3) / 1.0 (0.2) / 0.3 (0.5)	−0.1(0.3) / 0.3(0.2) / −0.5(0.4)	-1.4(0.2) / −7(0.2) / −2(0.4)	0.6 (0.2) / 0.9 (0.2) / 0.3 (0.8)	0.03 (0.2) / 1.3 (0.2) / −2.2 (0.3)
Rotamer / Cβ out (%)	3.8 / 0	4.8 / 0	2.0 / 0	4.1 / 0	3.6 / 0
Cis / Twisted proline	0 / 0	0 / 0	0 / 0	0 / 0	0 / 0
CaBLAM outliers (%)	0.4	3.8	2.1	2.2	5.3
Box Lengths (Å)	89.2/ 90.7/ 111.5	95.9/ 89.8/ 114.2	89.2/ 90.2/ 115.1	84.3/ 92.8/ 112.0	121.1/ 92.9/ 106.3

## Data Availability

Atomic coordinates for human importin β:αIBB, importin β:xIBB, importin β:αIBB:FG, importin β:Ran-GTP, and importin β:Ran-GTP:RanBP1 have been deposited in the Protein Data Bank with accession codes 9N86, 9N87, 9BFC, 9BAW, and 9N85. The cryo-EM density maps have been deposited in the Electron Microscopy Data Bank with accession codes EMD-49116, EMD-49117, EMD-44492, EMD-44412, and EMD-49114.
